# Levosimendan in the light of the results of the recent randomized controlled trials: an expert opinion paper

**DOI:** 10.1186/s13054-019-2674-4

**Published:** 2019-11-29

**Authors:** Bernard Cholley, Bruno Levy, Jean-Luc Fellahi, Dan Longrois, Julien Amour, Alexandre Ouattara, Alexandre Mebazaa

**Affiliations:** 1 Department of Anesthesiology and Critical Care MedicineP, Hôpital Européen Georges Pompidou, AP-HP, 20 rue Leblanc, 75015 Paris, France; 2 Université Paris Descartes - Université de Paris, Paris, France; 3 INSERM UMR_S1140, Paris, France; 4 CHRU Nancy, Réanimation Médicale Brabois, Vandoeuvre-les Nancy, France; 5 Department of Anesthesiology and Critical Care, Hôpital Cardiologique Louis Pradel, Lyon, France; 6 INSERM U1060, University Claude Bernard, Lyon, France; 7 Department of Anesthesiology and Critical Care, Hôpital Bichat-Claude Bernard, AP-HP, Paris, France; 8 Université Paris Diderot, Sorbonne Paris Cité, Paris, France; 9 Department of Anesthesiology and Critical Care Medicine, Hôpital de La Pitié Salpêtrière, AP-HP, Paris, France; 10 University Pierre & Marie Curie, Paris, France; 11 Department of Anesthesiology and Critical Care, Magellan Medico-Surgical Center, Bordeaux, France; 12 University of Bordeaux, Bordeaux, France; 13 INSERM, UMR 1034, Biology of Cardiovascular Diseases, Bordeaux, France; 14 Department of Anesthesia, Burn and Critical Care, Hôpitaux Universitaires Saint Louis Lariboisière, AP-HP, Paris, France

**Keywords:** Levosimendan, Catecholamine, Beta-blocker, Heart failure, Cardiogenic shock

## Abstract

Despite interesting and unique pharmacological properties, levosimendan has not proven a clear superiority to placebo in the patient populations that have been enrolled in the various recent multicenter randomized controlled trials. However, the pharmacodynamic effects of levosimendan are still considered potentially very useful in a number of specific situations.

Patients with decompensated heart failure requiring inotropic support and receiving beta-blockers represent the most widely accepted indication. Repeated infusions of levosimendan are increasingly used to facilitate weaning from dobutamine and avoid prolonged hospitalizations in patients with end-stage heart failure, awaiting heart transplantation or left ventricular assist device implantation. New trials are under way to confirm or refute the potential usefulness of levosimendan to facilitate weaning from veno-arterial ECMO, to treat cardiogenic shock due to left or right ventricular failure because the current evidence is mostly retrospective and requires confirmation with better-designed studies. Takotsubo syndrome may represent an ideal target for this non-adrenergic inotrope, but this statement also relies on expert opinion. There is no benefit from levosimendan in patients with septic shock. The two large trials evaluating the prophylactic administration of levosimendan (pharmacological preconditioning) in cardiac surgical patients with poor left ventricular ejection fraction could not show a significant reduction in their composite endpoints reflecting low cardiac output syndrome with respect to placebo. However, the subgroup of those who underwent isolated CABG appeared to have a reduction in mortality. A new study will be required to confirm this exploratory finding.

Levosimendan remains a potentially useful inodilator agent in a number of specific situations due to its unique pharmacological properties. More studies are needed to provide a higher level of proof regarding these indications.

## Introduction

Levosimendan was developed in the early 1990s in Finland and became available for prescription starting in 2001. It has been used since then in more than 60 countries. The unique pharmacological properties of this drug raised a major interest among physicians in charge of patients with heart failure, both in the medical and the surgical environments. Initial studies were most often positive, attesting for improvement in hemodynamics and/or organ function, and even suggested a reduced mortality [1, 2]. In 2007, the SURVIVE study, a large international multicenter randomized controlled trial (RCT), comparing levosimendan with dobutamine in patients with decompensated heart failure failed to show a difference between the two drugs on mortality at day 180 [3]. Shortly after, several metaanalyses suggested that levosimendan was able to reduce mortality in patients with poor left ventricular ejection fraction following cardiac surgery, placing this drug under the spotlight in the surgical environment [4–6]. But in 2017, three randomized controlled trials comparing levosimendan with placebo on top of standard care in cardiac surgical patients failed to reach statistical significance. Two of these studies addressed the question of the effectiveness of levosimendan as a prophylactic treatment (administration at the time of anesthesia induction: pharmacological preconditioning) for the prevention of postoperative low cardiac output syndrome (LCOS) in patients with poor left ventricular ejection fraction (LVEF) undergoing on-pump cardiac surgery [7, 8], and one study evaluated its effectiveness in reducing mortality at day 30 in patients developing post-bypass LCOS [9]. Thus, 2 years after the publication of the results of these studies, the place (if any) of levosimendan in our therapeutic armamentarium is questioned. The debate concerns the routine use of levosimendan to prevent and/or treat cardiac failure/dysfunction. The purpose of this opinion paper is to discuss the “non-routine” (or niche) indications of this drug according to the opinion of a group of expert users. Prior to discuss the situations in which levosimendan might still be considered an option, it is important to briefly remind the specific pharmacological properties of this agent as well as the potential detrimental effects of “classical” positive inotropic drugs.

## Pharmacological properties of levosimendan

Levosimendan has a pharmacodynamic profile combining inotropic and vasodilating effects (inodilator), and a nearly unique (among inotropes) myocardial protective effect. The inotropic effect results, in part, from an increased affinity of troponin C for calcium when the drug is present, which in turn prolongs the duration of actin/myosin cross-bridges [10–12]. This explains an increased contractility which is not associated with a raise in intracytoplasmic calcium concentration [13]. Therefore, this inotropic effect does not generate a direct significant increase in myocardial oxygen consumption, since most of the myocardial energy expenditure is related to the diastolic uptake of calcium in the sarcoplasmic reticulum [14, 15]. However, levosimendan and its metabolite have also been shown to inhibit phosphodiesterase III [16], an action that may participate to the positive inotropic effect via the c-AMP pathway, but that might, in turn, increase myocardial oxygen consumption. Levosimendan is a potent vasodilator through the opening of ATP-dependent potassium channels in vascular smooth muscles [17, 18]. It has been associated with a variety of myocardial protective effects against ischemia (preconditioning, postconditioning, anti-stunning, and anti-apoptotic effects) that are related to the opening of the same channels within the mitochondria of cardiac myocytes [19, 20], but the clinical evidence supporting these experimental findings is limited [21].

From the pharmacokinetic point of view, levosimendan has a fast onset of action and a half-life of 1 h. The drug undergoes hepatic metabolism (acetylation) followed by renal excretion. Quite uniquely, it has an active metabolite with a very long half-life (70–80 h) responsible for a prolonged effect [22, 23]. All these characteristics offer opportunities to provide an ideal therapeutic response to specific situations. Nevertheless, the longer half-life, as compared to catecholamines, may change the way clinicians manage side effects (e.g., arterial hypotension) and may sometimes prove inconvenient when quick reversibility is desirable. The most frequent adverse event associated with levosimendan administration is hypotension requiring norepinephrine infusion, although its prevalence was not significantly greater with respect to placebo [7–9] or dobutamine [3] in the largest RCTs published. In patients with septic shock, however, the cardiovascular dysfunction (reflected using the SOFA score) was more profound in patients receiving levosimendan [24]. These trials also found a greater proportion of patients receiving levosimendan developing atrial fibrillation, but the difference with the comparator group reached statistical significance only in medical heart failure and septic shock patients [3, 24].

## Potential detrimental effects of classical inotropic drugs

The “classical” inotropic drugs (catecholamines and phosphodiesterase III inhibitors) are widely used in the perioperative setting, particularly in patients undergoing cardiac surgery. While they can provide life-sustaining support in circumstances of severe right and/or left cardiac ventricular failure and improve both clinical symptoms experienced by patients and systemic end-organ perfusion, the benefits of these inotropes on medium- and long-term survival have never been documented [25]. To date, none of the available drugs satisfies all criteria of an ideal inotropic agent (Table 1), and there is no current evidence to recommend the choice of any of them over the others for the daily practice [26].

**Table 1:** Theoretical clinical characteristics of an ideal positive inotropic agent (from [25])

Easy titration for rapid on/off effect	
Myocardial oxygen supply/demand balance	
Steady effect in time (no tachyphylaxis)	
Direct positive inotropic effect	
β-independent positive inotropic stimulation	
Few or no arrhythmogenic effect	
No intracellular calcium overload	
Maintenance of the coronary perfusion pressure	
Beneficial effects on regional vascular beds	
Reasonable benefit/risk balance	

The catecholamines (especially dobutamine) are the most frequently used during the perioperative period. With an elimination half-life of just a few minutes, their on/off properties are quite appreciated at the bedside. The infusion of dobutamine produces a dose-dependent rise in cardiac output, mainly by increasing heart rate rather than stroke volume [27]. Major adverse cardiac events (ventricular arrhythmias and myocardial infarction), stroke, renal replacement therapy, and all-cause mortality (short- and long-term) increase when these agents are used during the perioperative period [28, 29]. Similarly, the use of catecholamines or phosphodiesterase III inhibitors in patients with acute heart failure is associated with an increase in mortality in the medical setting [30, 31]. The worsening in myocardial energy imbalance likely participates to the detrimental effects of the classical inotropic drugs, especially in situations of myocardial reperfusion injury [26]. However, many physicians still do not perceive the risk of complications associated with these drugs and keep using them routinely, sometimes despite a lack of evidence for their usefulness. It should also be clearly noted that levosimendan has not demonstrated a clear superiority to other inotropes in well-designed trials.

## Potential non-routine (niche) indications for levosimendan (Fig. 1)

### Levosimendan for heart failure patients receiving beta-blockers

Patients receiving beta-blockers (BB) in an acute or chronic setting have an altered beta-adrenergic receptor function and are therefore unlikely to respond optimally to catecholamines. The relevant clinical contexts are as follows: (i) patients with acute myocardial infarction given BB prophylactically who subsequently develop cardiogenic shock and require inotropic support, (ii) patients with chronic heart failure who are under chronic BB therapy and have acute on chronic cardiac decompensation, and (iii) patients with accidental/intentional BB intoxication. When beta-adrenergic receptors number and/or function are decreased, levosimendan appears as the drug of choice because its mechanisms of action are independent of this receptor. This is supported by a few experimental and clinical studies. In an animal model of acute intoxication with propranolol, Leppikangas et al. demonstrated that levosimendan, but not dobutamine or placebo, was able to increase stroke volume, inotropism, heart rate, and mean arterial pressure [32]. This was associated with a 100% survival of animals with levosimendan, as compared to 20% with dobutamine or placebo. In a small number (*n* = 52) of patients with acute or chronic heart failure and treated with beta-blockers, a prospective randomized double-blind international study found that levosimendan, as opposed to dobutamine, increased cardiac index and decreased pulmonary capillary wedged pressure, but failed to improve clinical symptoms and mixed venous oxygen saturation [33]. In addition, exploratory findings in favor of levosimendan were also reported from subgroup analyses of the LIDO and SURVIVE studies, comparing patients receiving beta-blockers versus those not receiving these drugs [1, 34]. Based on these possible beneficial hemodynamic effects, levosimendan is now considered the first-choice drug in patients with acute decompensated heart failure and on beta-blockers, if beta-blockade is thought to be contributing to hypotension with subsequent hypoperfusion (class IIb, evidence level C) [35, 36].

**Fig. 1:**
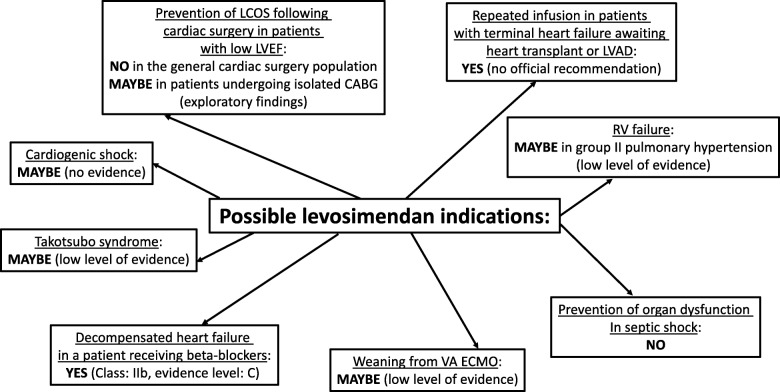
Potential indications for levosimendan. LCOS, low cardiac output syndrome; LVEF, left ventricular ejection fraction; CABG, coronary artery bypass graft; VA ECMO, veno-arterial extra-corporeal membrane oxygenation; LVAD, left ventricular assist device

### Repetitive infusions of levosimendan in patients with advanced chronic heart failure

Patients with symptomatic advanced heart failure despite optimal medical treatment are sometimes unable to be discharged because they are dependent on dobutamine infusions that cannot be weaned off. Such patients are at high-risk of death and may be waiting for a heart transplantation or a long-term mechanical assist device or, in contrast, may not be eligible for these therapeutic options. In such situations, repetitive infusions of levosimendan may offer the advantage of a prolonged inotropic effect with the possibility of improving the clinical symptomatology and allowing hospital discharge. Very limited data exists regarding the effectiveness of repetitive infusions of levosimendan [37–41]. A recent meta-analysis confirmed the potential usefulness of this approach, despite the heterogeneity of the existing studies [42]. The LevoRep study was the first multicenter RCT evaluating the effect of 4 injections of levosimendan (0.2 μg kg^−1^ min^−1^) over 6 h at 2-week intervals in 120 advanced heart failure patients [43]. The improvement in the 6-min walk test (primary outcome) was not significant, and the hazard ratio for the survival free of events was 0.50 (95% CI 0.25–1.05) favoring the group receiving levosimendan versus placebo (*p* = 0.069). The LION-HEART study, another multicenter RCT involving 69 patients with chronic advanced heart failure, compared the effect of 6-h levosimendan infusions (0.2 μg kg^−1^ min^−1^) repeated every 2 weeks for 12 weeks with placebo infusions on NT-proBNP levels at 12 weeks [44]. The authors observed that repetitive infusions of levosimendan significantly reduced the NT-proBNP, but also the readmission rate for acute heart failure at 12 months (20% versus 65%, *P* < 0.001) and attenuated the decline in quality of life (20% versus 63%, *P* = 0.022). The incidence of serious adverse events was not different between treatment and placebo groups, reflecting the safety of repetitive infusions of levosimendan. However, it is important to mention that although mortality and readmission rates decreased with levosimendan, they remained much higher in comparison to those observed when heart transplantation or mechanical circulatory assist devices could be carried out. Therefore, the strategy of repetitive infusions cannot be considered as an alternative to them, but alleviates the symptoms and improves the quality of life in patients awaiting transplantation or in those who are ineligible for more invasive approaches. A new RCT (LeoDOR, NCT03437226) is currently under way to try to confirm the usefulness of repeated infusions of levosimendan in this patient population [45].

### Levosimendan in cardiogenic shock due to left ventricular failure

Although the classical hemodynamic profile of cardiogenic shock associates low cardiac output, low arterial pressure, elevated left/right ventricular diastolic pressure, and elevated systemic vascular resistance, other phenotypes can be encountered. Some shock states related to ischemia-reperfusion injury lead to a sepsis-like syndrome with low systemic vascular resistance [46, 47], while others, on the contrary, present with maintained arterial pressure and signs of end-organ hypoperfusion due to a dramatic increase in systemic vascular resistance [48].

Unlike dobutamine, levosimendan increases moderately myocardial oxygen consumption, does not alter diastolic function, and has less direct pro-arrhythmic effects [49]. Moreover, as mentioned earlier, levosimendan acts independently of beta-adrenergic receptors activation and is therefore not sensitive to the action of beta-blockers. There are, however, potential difficulties to use levosimendan in cardiogenic shock. Levosimendan is also a potent vasodilator, which in the context of vasoplegic patients or in vasopressor-dependent patients might be associated to hypotension leading to increased vasopressor requirement. Finally, the very long half-life of levosimendan is a double-edged sword. This property is particularly interesting to wean the patient from catecholamines, but on the other hand, it may be difficult to rapidly reverse the vasodilation once the drug has been administered.

There is currently no high-quality study dealing with the use of levosimendan in cardiogenic shock. The most recent meta-analysis performed using a few studies with a high risk of bias, reports that, when compared to dobutamine, levosimendan did not affect short and long-term mortality, ischemic events, acute kidney injury, dysrhythmias, or hospital length of stay [50]. Levosimendan is often responsible for systemic arterial hypotension resulting in increased requirements for vasopressors, but also an increase in cardiac index and cardiac power, a decrease in left ventricular pressure, and an increase in SVO_2_ [51]. The need for randomized studies in this topic is strongly supported by two recent recommendations of the American Heart Association and the Cochrane collaboration [48, 50]. Currently, levosimendan is recommended as a “rescue” therapy in cardiogenic shock after dobutamine failure and before veno-arterial extracorporeal membrane oxygenation. The hypothesis to be tested is that the early use of levosimendan, by authorizing the discontinuation of dobutamine, would avoid the undesirable effects of catecholamines, accelerate the resolution of signs of low cardiac output, and facilitate myocardial recovery. One of the limits is the delay of action of levosimendan that can reach 2–5 h to produce an increase in stroke volume in the absence of a loading bolus. In patients having a postoperative low cardiac output syndrome following cardiac surgery, a large multicenter RCT failed to show a benefit of levosimendan on mortality [9]. A new RCT (LevoHeartShock, NCT04020263) is about to start to compare levosimendan versus placebo on top of conventional adrenergic inotrope therapy on a combined morbidity-mortality endpoint in patients with cardiogenic shock.

### Levosimendan in patients with Takotsubo syndrome

Takotsubo syndrome is a form of acute myocardial stunning in which catecholamines appear to have a central role in the pathophysiology, as there is no occlusive coronary artery disease to explain the pattern of temporary LV dysfunction observed. To date, there have been no randomized trials to define the optimal management of patients with suspected Takotsubo syndrome. Levosimendan has been advocated as the first choice inotropic support when mechanical circulatory assist devices are not available [52–54]. This approach is mainly supported by encouraging case reports and the pathophysiological rationale. Indeed, the use of adrenergic inotropes or phosphodiesterase inhibitors should be regarded as contraindicated in this situation as further activation of catecholamine receptors or their downstream molecular pathways might worsen the clinical status and prognosis of patients with Takotsubo syndrome and cardiogenic shock.

### Levosimendan in patients with pulmonary hypertension and right ventricular failure

There is experimental evidence attesting for the ability of levosimendan to reverse, in part, pulmonary vasoconstriction and improve right ventricular function in various animal models of pulmonary hypertension [55–58]. Patient data, however, is still very scant and heterogeneous, involving patients with various types of pulmonary hypertension and right heart failure, and often with contradictory results [59]. The largest set of data was obtained in patients with pulmonary hypertension and right ventricular failure as a consequence of severe LV failure (group 2) [60–63]. The benefit observed could be due to the effect of the drug on the pulmonary vasculature and the improvement in RV contractility or, alternatively, be secondary to the improvement in LV function resulting in less pulmonary congestion. Much less data is available on other forms of pulmonary hypertension (groups 1, 3, 4, and 5). Although attractive, the idea of using levosimendan to treat right ventricular failure should be considered very carefully: the potential benefits resulting from pulmonary vasodilatation and RV contractility improvement might be outweighed by the decrease in systemic arterial pressure and the subsequent reduction in right coronary perfusion pressure. An uncontrolled drop in myocardial oxygen delivery might precipitate right ventricular failure and result in acute cardiogenic shock.

### Levosimendan to facilitate weaning from veno-arterial extra-corporeal membrane oxygenation

Veno-arterial extra-corporeal membrane oxygenation (VA ECMO) is used to restore adequate perfusion to vital organs in patients suffering from refractory cardiogenic shock [64]. However, VA ECMO with femoral artery cannulation provokes an increase in left ventricular afterload while residual blood flow from the pulmonary and bronchial circulations keep flowing towards the left ventricle. Consequently, if the failing left ventricle is unable to eject against the retrograde flow of the VA ECMO, it may dilate and the resulting congestion may lead to pulmonary edema*.* To avoid this complication*,* low doses of positive inotropic drugs are commonly administered in order to maintain left ventricular ejection and avoid upstream congestion. Moreover, since the duration of VA ECMO is directly correlated to complications, the weaning of the device should be attempted as soon as possible. Although dobutamine is currently the first-line drug used for patients in cardiogenic shock [65, 66], the specific features of levosimendan are of interest in this situation [67–70]. However, despite an attractive rationale, only a few studies support the effectiveness of levosimendan to facilitate the weaning of VA ECMO and they are always retrospective [69, 71]. In addition, we have no clue regarding the ideal dosing and timing to initiate levosimendan infusion in patients suffering from cardiogenic shock and treated by VA ECMO. A multicenter randomized controlled trial is about to start to test the hypothesis that levosimendan in addition to standard care facilitates the weaning from VA ECMO.

### Levosimendan in patients with septic shock

Experimentally, in septic rabbits, levosimendan yields a similar improvement in left ventricular systolic function, when compared to dobutamine or milrinone, but improves diastolic function to a greater extent [72]. A large multicenter randomized controlled trial assessed the effect of levosimendan on organ dysfunction in adult patients with septic shock at day 28, but failed to show a reduction in organ dysfunction or mortality when added to standard care [24]. Moreover, patients who were assigned to receive levosimendan required more norepinephrine, were less likely to be weaned from mechanical ventilation, and had more atrial fibrillation. Furthermore, in a sub-group analysis of the LeoPARDS trial, Antcliffe et al. used the biomarkers cTnI and NT-proBNP to identify patients with evidence of myocardial injury and dysfunction, respectively. These authors did not observe any benefit from using levosimendan in any subgroup classified by a variety of biomarker cut-off thresholds [73]. No data suggests differences in outcome between dobutamine and levosimendan in septic shock patients. Levosimendan might potentially remain useful in case of septic shock patients admitted with beta-blocker therapies that could participate to their circulatory failure.

### Prophylactic Levosimendan in cardiac surgical patients with low LVEF

Although two large randomized controlled trials failed to show a reduction in composite endpoints reflecting low cardiac output syndrome and mortality in a mixed population of CABG, valvular, or combined surgery with LVEF< 40% [7, 8], a recent meta-analysis suggested that there could be a greater benefit in the isolated CABG population [74]. This exploratory finding is a strong incentive to re-evaluate the preoperative infusion of levosimendan prior to CABG surgery in patients with poor LVEF.

## Conclusion

Due to a very interesting pharmacological profile, levosimendan has raised a lot of interest in the field of heart failure management. Unfortunately, all the large randomized controlled trials have failed to demonstrate a clear superiority of this drug over placebo, when used on top of usual catecholamines, in the populations that they have tested. Even if the initial enthusiasm for this drug has somewhat been reconsidered, there are still good reasons to believe that it might be useful in specific subgroups. These include patients with acute heart failure receiving beta-blockers and Takotsubo syndrome, patients awaiting heart transplant or left ventricular assist device implantation, and patients under VA ECMO to facilitate weaning. In addition, there may still be room for levosimendan in the management of some patients with cardiogenic shock, and as a prophylactic treatment prior to CABG surgery in patients with low LVEF. Additional studies are still required to support these potential indications with a higher level of evidence.

## Data Availability

NA

## Abbreviations

BB: Beta-blocker
CABG: Coronary artery bypass graft
LCOS: Low cardiac output syndrome
LV: Left ventricle
LVAD: Left ventricular assist device
LVEF: Left ventricular ejection fraction
RCT: Randomized controlled trial
RV: Right ventricle
VA ECMO: Veno-arterial extra-corporeal membrane oxygenation
